# Daunorubicin, Cytarabine, and Cladribine Regimen Plus Radiotherapy and Donor Lymphocyte Infusion for Extramedullary Relapse of Acute Myeloid Leukemia after Hematopoietic Stem Cell Transplantation

**DOI:** 10.1155/2013/258028

**Published:** 2013-08-27

**Authors:** Marco Sanna, Giovanni Caocci, Adriana Vacca, Eugenia Piras, Federica Orrù, Giorgio La Nasa

**Affiliations:** ^1^Hematology Unit and Bone Marrow Transplantation Center, “R. Binaghi” Hospital, Via Is Guadazzonis 3, 09126 Cagliari, Italy; ^2^Department of Medical Sciences, University of Cagliari, 09100 Cagliari, Italy

## Abstract

Myeloid sarcoma is a rare tumor consisting of myeloid blasts that involve anatomic sites outside the bone marrow. Fatal prognosis is inevitable in patients with extramedullary relapse after hematopoietic stem cell transplantation (HSCT), and no standard treatments are available yet. We report the first case of extramedullary relapse after HSCT treated with a combination of daunorubicin, cytarabine, and cladribine (DAC) regimen plus radiotherapy and donor lymphocyte infusion (DLI). This treatment induced a new and durable remission in our patient. The favorable toxicity profile and the reduced cost make this combination worthy of further investigations.

## 1. Introduction

Myeloid sarcoma is a rare tumor consisting of myeloid blasts that involve anatomic sites outside the bone marrow [1]. It may present “de novo,” before or concurrently with the diagnosis of acute myeloid leukemia (AML) or at relapse, with or without bone marrow involvement [2, 3]. The incidence of myeloid sarcoma in patients who underwent allogeneic bone marrow transplantation for AML is low and varies from 0.2 to 1.3% [4]. The prognosis of transplanted patients presenting extramedullary relapse is poor, and there are no standardized recommendable treatments. The various available therapy approaches include infusion of donor lymphocytes (DLI), radiotherapy (RT), chemotherapy, or experimental drugs [3]. 

## 2. Case Presentation

Here is reported the case of a 29-year-old patient, diagnosed in November 2009 with AML, carrying the cytogenetic translocation t(6 : 11). The patient received an induction treatment, based on standard cytarabine plus daunorubicin (7 + 3) therapy. Due to the persistence of 30% blasts in the bone marrow, a reinduction with the fludarabine, cytarabine, and idarubicin (FLAI) schedule was performed. Unfortunately, 25% of blasts were still present. In February 2010, a cycle of salvage chemotherapy based on clofarabine, high-dose cytarabine plus gemtuzumab ozogamicin was administered, and the patient achieved a good hematologic remission, showing a minimal residual disease (MRD) by the immunophenotypic analysis, with only 0.2% of myeloid blasts. A new cycle with the same schedule was repeated, and the patient achieved a complete remission with no evidence of MRD. As an HLA-identical unrelated donor was available, she underwent hematopoietic stem cell transplantation (HSCT) in June 2010, following a conditioning regimen based on busulfan and cyclophosphamide. Graft versus host disease (GvHD) prophylaxis consisted of cyclosporin and short courses of methotrexate. Complete allogeneic engraftment was achieved, and MRD was confirmed negative afterwards. Unfortunately, after 2 years from HSCT, the patient noticed a painful swelling in the left breast. A percutaneous biopsy of the lesion showed the presence of a myeloid sarcoma; the patient was restaged, and examinations confirmed no MRD at bone marrow level. A positron emission tomography (PET) scan confirmed the presence of lesions with high metabolic activity both in the right breast and in the left breast. We decided to treat the patient with an integrated approach including RT, chemotherapy, and immunotherapy. Firstly she was treated with RT 24 Gy directed to breast lesions. Subsequently, she underwent two courses of DAC regimen, in order to modify the natural history of the myeloid sarcoma that results in the relapse at bone marrow level in most cases. The therapy was well tolerated, with no complications. Disease reevaluation by the PET scan showed the absence of lesions with high metabolic activity. In order to consolidate the result, the patient underwent DLI every 5 weeks, with CD3+ lymphocyte dose progressively increased up to 1 × 10^7^/kg. Currently the patient is well with no active lesions at the PET scan. 

## 3. Discussion

Fatal prognosis is inevitable in patients with extramedullary relapse after HSCT, and no standard treatments are available yet [3]. At the moment new therapeutic protocols are therefore necessary. Cladribine proved to be effective in combination with cytarabine and doxorubicin in the treatment of newly diagnosed and relapsed AML [5–7]. Cladribine causes a direct mitochondrial damage and exerts its toxicity even on nonactively proliferating cells [8]. To our knowledge, our report is the first on extramedullary relapse after HSCT treated with a combination of DAC regimen, RT, and DLI. This treatment induced a new and durable remission in our patient, previously refractory to several lines of chemotherapy. The favorable toxicity profile and the reduced cost make this combination worthy of further investigations in this setting of high-risk patients. 
